# Corrosion Resistant FBG-Based Quasi-Distributed Sensor for Crude Oil Tank Dynamic Temperature Profile Monitoring

**DOI:** 10.3390/s151229811

**Published:** 2015-12-04

**Authors:** Rogério da Silva Marques, Adilson Ribeiro Prado, Paulo Fernando da Costa Antunes, Paulo Sérgio de Brito André, Moisés R. N. Ribeiro, Anselmo Frizera-Neto, Maria José Pontes

**Affiliations:** 1 Federal Institute of Espírito Santo, Av. Filogônio Peixoto 2220, Bairro Aviso, Linhares 29901-291, Brazil; marquesrs2@gmail.com; 2 Federal University of Espírito Santo, Department of Electrical Engineering, Av. Fernando Ferrari, 514, Goiabeiras, Vitória 29075-910, Brazil; adilsonp@ifes.edu.br (A.R.P.); moises@ele.ufes.br (M.R.N.R.); frizera@ieee.org (A.F.-N.); 3 Federal Institute of Espírito Santo, Rodovia ES 010, km 6.5-Manguinhos, Serra 29173-087, Brazil; 4 Instituto de Telecomunicações, I3N and Physics Department, University of Aveiro, Campus Universitário de Santiago, Aveiro 3810-193, Portugal; pantunes@av.it.pt; 5 Instituto de Telecomunicações and Department of Electrical and Computer Engineering, Superior Technical Institute , University of Lisbon, Av. Rovisco Pais, Lisbon 1049-001, Portugal; paulo.andre@lx.it.pt

**Keywords:** FBG, crude oil, PTFE, temperature

## Abstract

This article presents a corrosion resistant, maneuverable, and intrinsically safe fiber Bragg grating (FBG)-based temperature optical sensor. Temperature monitoring is a critical activity for the oil and gas industry. It typically involves acquiring the desired parameters in a hazardous and corrosive environment. The use of polytetrafluoroethylene (PTFE) was proposed as a means of simultaneously isolating the optical fiber from the corrosive environment and avoiding undesirable mechanical tensions on the FBGs. The presented sensor head is based on multiple FBGs inscribed in a lengthy single mode fiber. The sensor presents an average thermal sensitivity of 8.82 ± 0.09 pm/°C, resulting in a typical temperature resolution of ~0.1 °C and an average time constant value of 6.25 ± 0.08 s. Corrosion and degradation resistance were verified by infrared spectroscopy and scanning electron microscopy during 90 days exposure to high salinity crude oil samples. The developed sensor was tested in a field pilot test, mimicking the operation of an inland crude tank, demonstrating its abilities to dynamically monitor temperature profile.

## 1. Introduction

Temperature monitoring is a fundamental problem in the oil and gas industry, especially for crude oil, since fluid temperature affects volumetric efficiency, viscosity, and density. Therefore, accurate and reliable temperature measurements are paramount all along the production chain; namely during oil extraction, transportation, storage, and refining [1,2]. For the production and transport operations, global standards impose that for custody transfer, trading, and taxing purposes, oil volumes should to be converted to its equivalent at 20 °C [3]. Thus, temperature inaccuracies at this stage will be directly translated into monetary damage for companies, individuals, and governments. 

Moreover, it is widely known that all oil production workplaces (*i.e.*, the environment, the equipment and the process) impose severe operational restrictions, owing to its explosive, toxic, and corrosive nature; not to mention the remoteness of oil production fields. On the other hand, those challenges bring along opportunities, since small innovations in the oil industry can generate large economic returns [4]. 

Any temperature measurement system designed for this industry should meet criteria such as: (i) long durability in chemically-hostile and hazardous environments; (ii) simple calibration; (iii) ease of operation; (iv) reduced time response; and (v) intrinsic safety. 

Temperature measurement can be made by different techniques such as: (i) thermographs infrared systems [5]; (ii) thermometer based on semiconductor film [6]; and acoustic measurement methods [7]. However, industrial processes in oil production heavily rely on platinum resistance temperature detectors, usually named as Pt100, with a typical resistance of 100 Ω at 0 °C [8]. 

A sensing head based on Pt100 suited for crude oil temperature measurements should meet explosion proof standards, which usually leads to cumbersome (high mass) and stiff devices with compromised features related to response time and maneuverability [9,10]. For their particular application in storage tanks, intrinsic safety is somehow compromised by having live wires connections at the top of tanks, where inflammable vapors accumulate. Moreover, frequent recalibration is also required, leading to time consuming, laborious and costly transport operations.

An appealing solution for scenarios where traditional electrical sensors are handicapped is the one based on optical fibers. With small footprints, the lightweight optical sensors can withstand harsh environments, and most importantly, are truly intrinsically safe due to the lack of electrical parts or connections. In addition to providing highly reliable measurements, the interrogating unit can be far apart (usually dozen of km or more) from the sensing point and can gather multiple sensor readings at once either through distributed [11] or concentrated optical sensing methods [12]. 

An increased number of publications regarding optical fiber sensors based on fiber Bragg gratings FBG or Fabry-Perot cavities [13] have been reported lately in the literature for different applications [14,15,16]. The improvement in FBG sensor’s performance to monitor parameters such as temperature and pressure increased its applications in the oil and gas industry. The development of new materials and techniques to enhance the FBG resilience to harsh environments has also been recently reported [17]. 

Alternatively, distributed optical sensors allow monitoring temperature, strain and vibration from any point along an optical fiber through light backscattering. The performances of distributed temperature sensors are suitable for many applications that require large areas of coverage with high location accuracy [11]. Distributed sensor commonly explores effects of Rayleigh and high power induced effects such as Brillouin and Raman scattering [18], hybrid solutions, even including FBGs, are also reported [19]. Compared with fiber based distributed temperature sensors, our proposed sensor based on FBG has the advantage of being intrinsically safe with spatial resolution compatible with the dimensions of the crude oil tanks. 

In this work we exploit the capabilities of an FBG-based sensor for multipoint sensing on a single fiber. The proposed innovative design for the sensor head allows the operation directly in contact with the crude oil, for temperature measurements of inland production tanks. Polytetrafluoroethylene (PTFE) coating was added for mechanical and chemical protection. PTFE’s corrosion and degradation resistance were verified by infrared spectroscopy and scanning electron microscopy. Section 2 presents the sensor description and the evaluation of the robustness to stand the chemically aggressive environment is presented in Section 3. The sensing element production and the sensor characterization are found in Section 4, whereas a field pilot test is presented in Section 5. The paper final remarks are given in Section 6. 

## 2. Sensor Constructive Description

**Figure 1:**
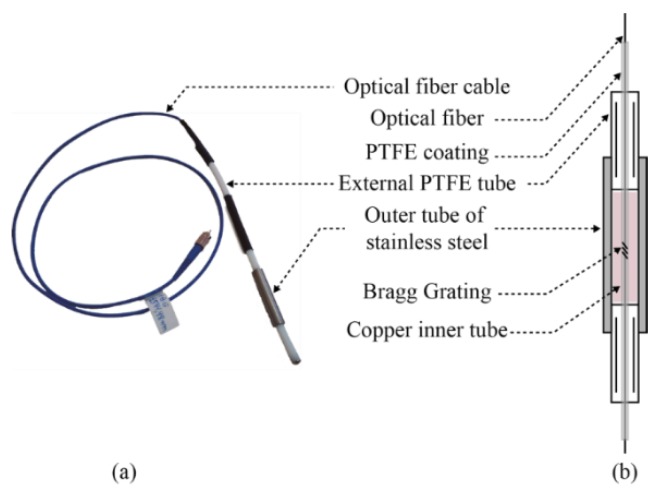
(**a**) Photography of the prototype with one sensing element; (**b**) Scheme of the FBG sensing element.

The optical fiber was left loose in two concentric PTFE tubes, the first one with 0.5 mm and the second one with 6.35 mm of external diameter. This is necessary to add chemical and mechanical strength to the sensor, while also avoiding mechanical tensions on the temperature sensing element. A stainless steel tube is used, only at the FBG fiber section, to increase the thermal conductivity between the sensing element and the outside medium. An anchor (not shown in Figure 1 and addressed in the field pilot section) was threaded onto the sixth sensing element in order to prevent (or at least minimize) the movement of the fiber sensor in the crude oil tanks during their operation cycles. Figure 1a depicts an initial prototype with just one sensing element and a detailed schematic representation of that sensing element is in the sensor head in Figure 1b. A copper inner tube, concentric with the stainless steel tube, was also tested within the FBG section for improving thermal conductivity. The connections between PTFE and stainless steel tubes are accomplished by mechanical threads made directly on these materials and sealed with PTFE-based adhesive LOCTITE^TM^ 5113.

According to the operational requirements of the oil industry, temperature measurement in crude oil tanks should be performed at least in three different points at different heights [20]. In our case, six different sensing points were considered to enable the monitoring, with high resolution, of the temperature gradient inside the crude oil tank. Moreover, this setup can easily be incremented to include more sensing points. The proposed quasi-distributed sensor is based on multiple evenly spaced FBGs, inscribed on a single mode optical fiber as will be described in Section 4, but before using these elements for temperature profiling, the overall sensor inertness should be tested. 

## 3. Inertness Test for High Salinity Crude Oil

The saline nature of crude oil promotes the degradation of polymeric materials as reported in studies concerning the aging of polymers [21,22]. Another major concern is that petroleum is a mixture of solvents which causes the degradation of most polymers, as discussed in [23]. Biological processes also may promote the degradation of a polymeric material as presented in [24]. Therefore, studies of corrosion and diffusion were accomplished by immersing PTFE test samples in high salinity crude oil, for testing the material physical and chemical degradation. 

The chemical corrosion analysis, as studied in this work, gives reliable information regarding the durability of PTFE in crude oil environment. Unlike processes used for the electrochemical monitoring of metals, polymer corrosion action should be evaluated from the point of view of chemical degradation, thus infrared spectrometry is the primary tool used in our analysis. The PTFE corrosion or chemical degradation analyses, applying infrared spectrometry, were previously explored in [25,26]. In addition, the technique of scanning electron microscopy (SEM) allows the analysis of the surfaces evolution, after direct contact with crude oil samples. 

The evaluation of the corrosion effect on the PTFE was performed with high salinity crude oil, basic sediment and water (BSW) level lower than 0.05% and total acidity of 0.31 mg KOH/g [27]. PTFE samples were submitted to a long oil exposure carried out during a 90 day period; this interval was conveniently selected since this is the time frame used for the chemical monitoring of oil storage systems [28]. During the tests individual samples were taken every week from the oil for characterization purposes. PTFE samples were washed with toluene, used only to remove the excess oil in contact with the polymer. Then, the samples were washed with a bath of surfactant and water.

**Figure 2:**
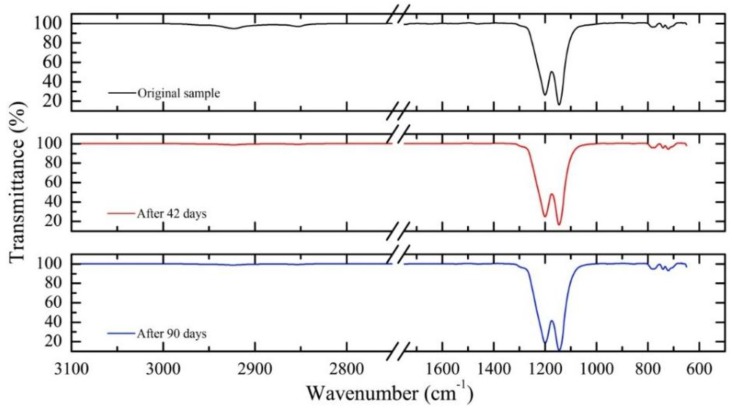
Transmission infrared spectroscopy for PTFE samples immersed in oil: initial (**top**), after 42 days (**middle**) and after 90 days (**bottom**).

Figure 2 shows the PTFE samples’ transmittance, as a function of wavelength (actually wavenumber as the usual unit in these tests), for three representative periods. PTFE transmittance was not significantly affected by the exposure to crude oil, even for those maintained immersed in oil up to 90 days. A sample not exposed to the oil was taken as reference. The absorption band suggests there is no change in the material composition after immersing in crude oil, meaning that the functional groups of the polymer under study remain stable. The analysis shows no evidence of chemical degradation or impregnation by crude oil components. 

**Figure 3:**
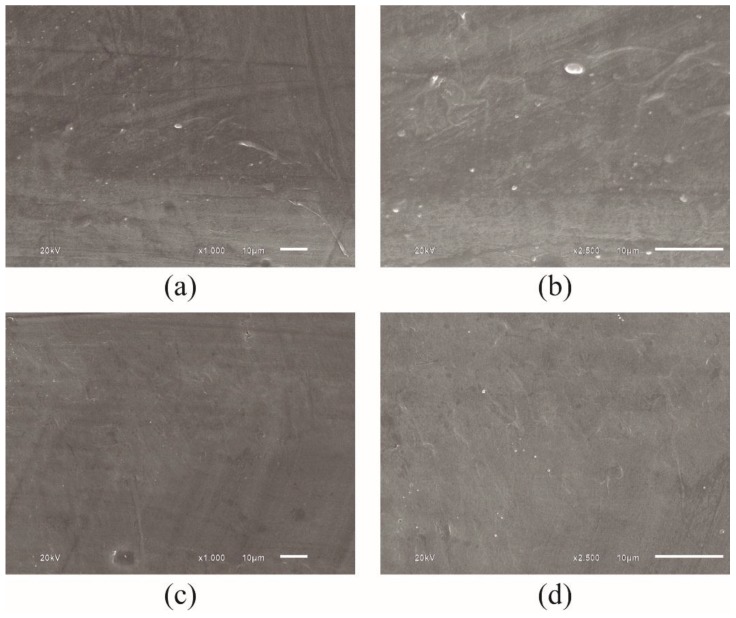
PTFE samples under different magnification. (**a**) PTFE reference with magnification of × 1000; and (**b**) PTFE reference with magnification of × 2500 times; (**c**) PTFE sample immersed in oil for 90 days, with magnification of × 1000; and (**d**) PTFE sample immersed in oil for 90 days, with magnification of × 2500 times.

The PTFE samples were also scanned by SEM in order to verify any superficial morphological irregularities caused by crude oil degradation. The visual analysis of the images obtained by SEM presented similar textures, indicating no degradation or impregnation by crude oil. As it is clearly observed in Figure 3, regardless of the magnification used in the images, the sample remaining 90 days in contact with the oil samples showed identical characteristics to those not exposed to the crude oil bath. The minor morphological differences present in the samples can be attributed to the manufacturing process. 

These results support that the inert behavior from PTFE suits the crude oil shielding needed in our sensor prototype. In addition, PTFE stands a wide temperature range (typically from −73 °C to 204 °C) and its excellent thermal and electrical insulation properties contribute to the intrinsic safety features of our sensor head. Finally, the low coefficient of friction presented by PTFE allowed us, as presented in Figure 1b, to use a second PTFE tube protecting the sensing element. Thus, this combination not only reduces mechanical tensions that can affect any FBG-based sensor precision, but also provides a (contiguous) second layer of protection to the optical fiber. This hermetic barrier will isolate the sensing element from direct contact with crude oil in an eventual infiltration of oil through the connection between the outer stainless still tube and the external PTFE.

## 4. Sensing Element Production and Sensor Characterization

The inscription of the Bragg gratings used in this work was made on single mode photosensitive commercially available optical fiber (FiberCore PS1250/1500), using the phase mask method with an excimer (KrF) laser emitting at 248 nm. For each inscribed FBG, the exposure time was 15 s, with a pulse energy of 5 mJ and a frequency of 500 Hz. All the FBG were inscribed with a wavelength increasing, in the same fiber strand, which was spliced to a standard single mode silica fiber. The multiplexed fiber strand was then introduced into a first protective PTFE tube as previously described. 

**Figure 4:**
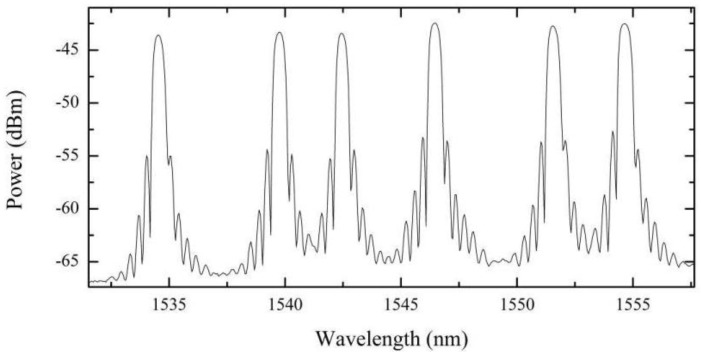
Optical reflection spectrum of the temperature sensor with six FBGs.

Figure 4 shows the optical reflection spectrum for the multiplexed FBGs, measured at 22 °C, attained with the Micron Optics model SM125 interrogator. The peak wavelengths of the FBGs are centered at 1534.814, 1539.995, 1542.735, 1546.728, 1551.820 and 1554.916 nm.

**Figure 5:**
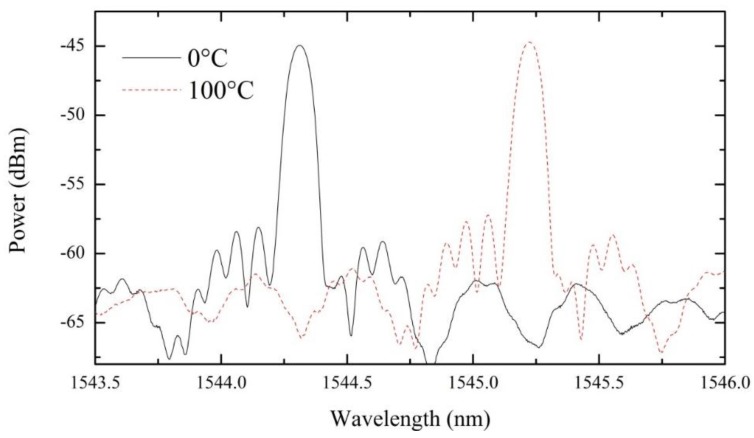
Reflection optical spectra for one FBG at 0 °C and 100 °C.

The prototype temperature calibration was made with conventional calibration procedure used in the oil industry; a dry block high performance calibrator (Presys model T25N), with a reference thermometer (Presys, model ST-501). This calibration system with external super thermometer allows 0.05 °C accuracy. Moreover, to improve the thermal conduction and temperature homogenization in the sensor region, small (~1 mm) metal spheres were used to position the prototype in a metallic cylinder adapted to the oven tube. The prototype response was attained for increasing and decreasing temperature cycles, from 0.0 °C to 100.0 °C in steps of 10.0 °C. This procedure was repeated 10 times for all the FBGs and the average values considered. No thermal hysteresis was observed in the prototype experimental characterization. For illustration purposes, Figure 5 displays the reflection optical spectra of one FBG, for two extreme temperatures of our tests.

**Figure 6:**
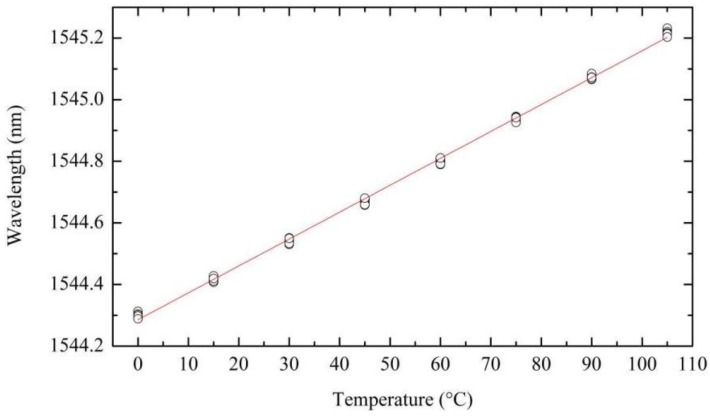
FBG peak wavelength as a function of temperature, the circles show the experimental data and the line is the linear fit over the measurement points.

The peak wavelength for a free standing FBG is given by:

(1) $$\lambda \left(T\right)=k_{t}\times T+\lambda_{0}$$

where λ is the wavelength, λ*_0_* corresponds to the FBG wavelength peak at 0 °C, *k_t_* is the thermal sensitivity and *T* is the temperature (in ° Celsius). The linear fitting of the FBG peak wavelength as a function of temperature, as shown in Figure 6 for one FBG, yields a thermal sensitivity value of 8.73 ± 0.027 pm/°C. 

The sensor with six FBGs present an average thermal sensitivity of 8.82 ± 0.09 pm/°C per FBG at 22 °C, with individual sensitivities of 8.82 ± 0.05, 8.56 ± 0.08, 8.71 ± 0.11, 8.81 ± 0.14, 8.98 ± 0.07 and 9.01 ± 0.09 pm/°C, respectively, for FBG1 through FBG6. This results in a typical temperature resolution of ~0.1 °C and an average time constant value of 6.25 ± 0.08 s. 

**Figure 7:**
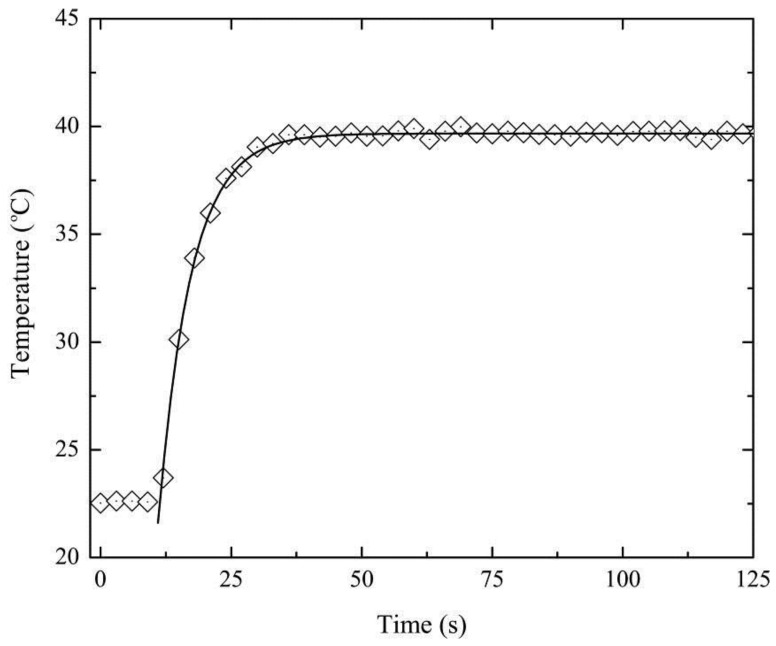
FBG based temperature sensor response as function of time when subjected to a temperate step: The diamonds show the experimental data and the line is the fitting.

The complete temporal response was characterized imposing a temperature step, by suddenly immersing it in stable water bath (at 40 °C). The Bragg wavelength and consequently the temperature, was registered every second, and displayed in Figure 7. Once again, for simplification, it is only shown the results for one FBG.

The measured thermal variation is given, considering the Newton's law of cooling, by:

(2) $$T\left(t\right)=T_{e}+\left(T_{0}-T_{e}\right)\times e^{\left(-k\times \left(t-t_{0}\right)\right)}$$

where *T_0_* is the initial temperature, *T_e_* corresponds to the final temperature and *k* is the constant of proportionality accounting the sensor head mass, heat capacity and thermal conductivity. The fitting of the experimental data to Equation (2) allows us to calculate k = 0.160 ± 0.002 s^−1^. This value indicates a sensor time constant of 6.25 ± 0.08 s.

In order to give a reference for the performance of the sensor time constant, one of the fastest Pt100 based thermometers available for laboratory use (with a very fragile protective tube of 3.2 mm) presents a time constant of tens of seconds [29]. It is needless to mention that industrial applications in crude oil would require better physical protection compared with these lab probes. Protection stilling wells are often employed to isolate the Pt100 sensors from the corrosive oil, and such apparatus can easily take time constants of conventional sensors to hundreds of seconds. This will often limit Pt100 use in dynamic temperature profile monitoring.

## 5. Field Pilot Test

**Figure 8:**
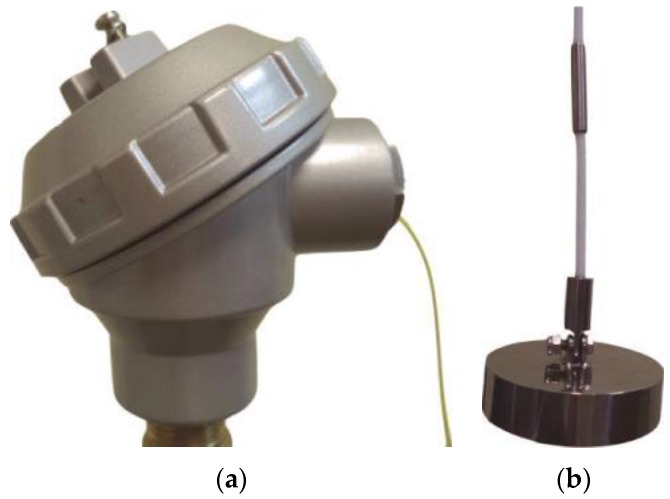
Photo of the (**a**) mounting head; and (**b**) anchor attached to the sensor.

A field pilot test with the quasi-distributed temperature sensor employing six FBGs was done recurring to a water reservoir equipped with an electric heater. Figure 8 shows the sensor head and the anchor attached to the PTFE outer coating by internal thread. To facilitate the installation of the sensor in the pilot oil tank, a mounting head was adapted allowing the easy connection to the fiber optics (Figure 8a. Additionally, an anchor was attached to the lower extremity of the sensor system to improve its mechanical stabilization, as depicted in Figure 8b.

**Figure 9:**
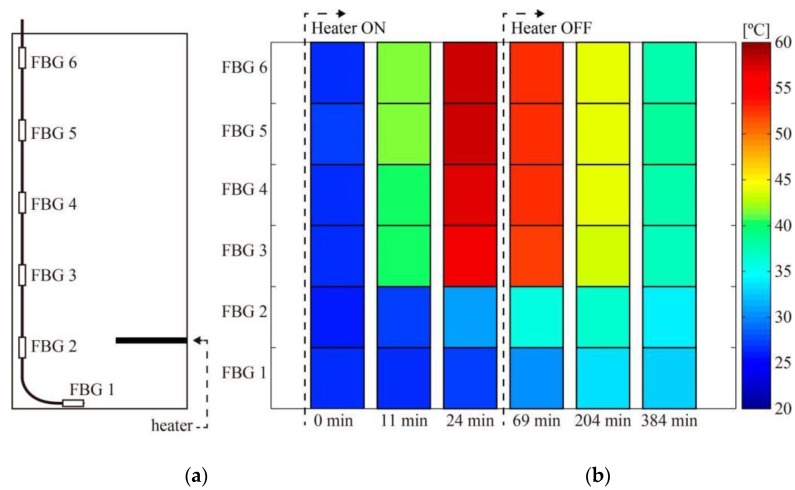
In-scale pilot representation of the oil tank. (**a**) Illustration of the fiber sensor immersed in the oil tank; (**b**) The quasi-distributed temperature profile.

This test emulates a crude oil tank operation with reduced flow. To simulate a scenario with high temperature oil injection at the tank base level, a 6000 W electrical heater was included. In the reservoir, with a capacity of, approximately, 100 liters the sensors were placed near the tank lateral wall and one located in the base near the tank center, as illustrated in Figure 9a. The temperature acquisition was performed using the SM125 interrogator (Micro Optics), with acquisition every minute during the heating process and every fifteen minutes during cooling. The objective of the heating process consisted on elevating the fluid temperature from room temperature to approximately 60 °C, which is a typical temperature in field operation.

The temperature measurements during heating and cooling are shown in terms of temperature profile perspective in Figure 8. Since the FBGs are immersed in a large volume, each one senses a different temperature over time, depending on its position inside the tank as no mechanism to homogenize the liquid temperature was employed. Convective heat transfer effects are clearly observed in the experiment, with FBG #5 measuring higher temperatures 24 min after heating in the water inside the tank, whereas FBG #1 detected lower temperatures during the temperature monitoring interval. 

**Figure 10:**
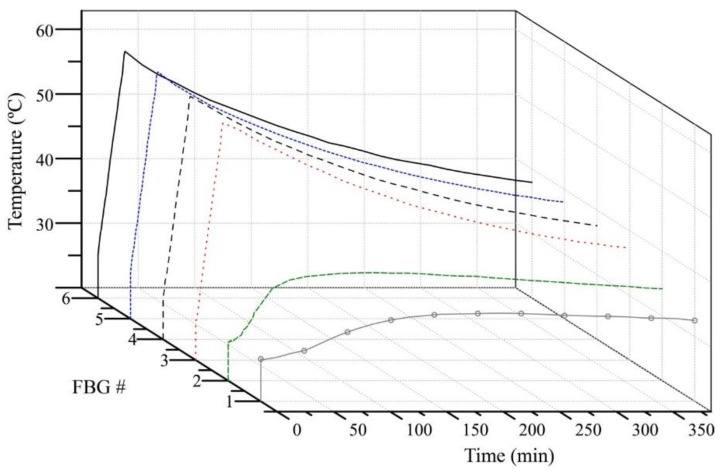
Temperature evolution along heating and cooling cycles for the quasi-distributed temperature sensor.

In order to give a better perspective of the heated fluid dynamics, Figure 10 shows the temperature profile in the water tank along the time. Since FBG1 is at the bottom of the water reservoir (below the heating element), it experiences a lower temperature increase than the FBGs that are above the heater due to the convective heat transfer. In addition, the temperature dynamics in different points inside the tank is quite inhomogeneous, as observed in Figure 10 for FBGs #1 and #6, for instance.

The temperature pattern shown in Figure 10 features the expected behavior for heating and cooling in the liquid reservoir, which is determined according to the dynamics of currents liquid convection. The sensor system has, therefore, shown the capacity for dynamic temperature profile monitoring.

## 6. Conclusions

This work presented a temperature measuring device based on FBG technology for operating immersed in crude oil. Multiple sections of FBG inscribed in a lengthy single mode fiber were inserted into a stainless steel tube to enhance the thermal conductivity, and protect it from the corrosive action of the environment. The sensor, containing six FBGs, has shown an average value for thermal sensitivity of 8.82 ± 0.09 pm/°C. The sensor device operates with sub miliwatt optical power, thus being fully applicable in explosive industrial environments. The temperature variation in terms of the prototype time response was characterized, presenting a thermal sensitivity value of 8.73 ± 0.027 pm/°C.

The polymer (PTFE) chosen for protecting the FBG is inert and offers corrosion resistance, excellent mechanical strength and can stand high temperature environments. It makes possible the direct immersion of the sensor in crude oil. No degradation was detected during 90 days by infrared spectrometry and scanning electron microscopy. Finally, in a pilot test, the device has shown great potential to be used in direct contact with crude oil for proper dynamic temperature profile monitoring. This will unveil the complex multiphase fluid thermodynamic effects in crude oil tanks. The next step will be the long-term field test of the sensor in actual inland crude oil tanks for checking performance under real operational conditions. Future works will also address the combination of space and spectral distribution for denser sensor setup with different materials for coating, evaluating their impact on the sensor time response.
